# microRNA regulation of skin pigmentation in golden-back mutant of crucian carp from a rice-fish integrated farming system

**DOI:** 10.1186/s12864-023-09168-w

**Published:** 2023-02-10

**Authors:** Xianbo Zhang, Mingkun Luo, Bingjie Jiang, Wenbin Zhu, Qianwen Min, Jinli Hu, Ting Liu, Jianjun Fu, Xiulan Shi, Pan Wang, Lanmei Wang, Zaijie Dong

**Affiliations:** 1 Guizhou Fisheries Research Institute, Guizhou Academy of Agriculture Sciences, Guiyang, Guizhou, China; 2 Key Laboratory of Freshwater Fisheries and Germplasm Resources Utilization, Freshwater Fisheries Research Center of Chinese Academy of Fishery Sciences, Ministry of Agriculture and Rural Affairs, Wuxi, Jiangsu, China; 3 Wuxi Fisheries College, Nanjing Agricultural University, Wuxi, Jiangsu, China; 4 College of Fisheries and Life Science, Shanghai Ocean University, Shanghai, China

**Keywords:** miRNA, Skin color, Golden-back crucian carp, miR-196d, *Myh*7

## Abstract

**Background:**

MicroRNAs (miRNAs) are endogenous small non-coding RNAs (21–25 nucleotides) that act as essential components of several biological processes. Golden-back crucian carp (GBCrC, *Carassius auratus*) is a naturally mutant species of carp that has two distinct body skin color types (golden and greenish-grey), making it an excellent model for research on the genetic basis of pigmentation. Here, we performed small RNA (sRNA) analysis on the two different skin colors via Illumina sequencing.

**Results:**

A total of 679 known miRNAs and 254 novel miRNAs were identified, of which 32 were detected as miRNAs with significant differential expression (DEMs). 23,577 genes were projected to be the targets of 32 DEMs, primarily those involved in melanogenesis, adrenergic signaling in cardiomyocytes, MAPK signaling pathway and wnt signaling pathway by functional enrichment. Furthermore, we built an interaction module of mRNAs, proteins and miRNAs based on 10 up-regulated and 13 down-regulated miRNAs in golden skin. In addition to transcriptional destabilization and translational suppression, we discovered that miRNAs and their target genes were expressed in the same trend at both the transcriptional and translational levels. Finally, we discovered that miR-196d could be indirectly implicated in regulating melanocyte synthesis and motility in the skin by targeting to *myh*7 (myosin-7) gene through the luciferase reporter assay, antagomir silencing in vivo and qRT-PCR techniques.

**Conclusions:**

Our study gives a systematic examination of the miRNA profiles expressed in the skin of GBCrC, assisting in the comprehension of the intricate molecular regulation of body color polymorphism and providing insights for *C. auratus* breeding research.

**Supplementary Information:**

The online version contains supplementary material available at 10.1186/s12864-023-09168-w.

## Background

MicroRNAs (miRNAs) are small, single-stranded, endogenous non-coding RNA molecules containing 21 to 23 nucleotides [1]. Mature miRNAs are produced from longer primary transcripts that are sheared and processed by a series of nucleases and subsequently assembled into an RNA-induced silencing complex (RISC) that recognizes the 3′-untranslated regions (UTRs) of the target mRNA, thereby inducing cleavage or blocking translation of the gene [2]. Recent studies have demonstrated that miRNA-mRNA interactions are significantly related to the transcriptional and signal transduction events involved in a wide range of biological processes, including apoptosis, cell proliferation, embryo development and skin pigmentation [3–5]. Via Illumina sequencing, Wang et al. [6] found that miR-107b and miR-141-3p were up-regulated in pink/black and pink/red skin of red tilapia (*Oreochromis* spp.) compared to whole pink skin. Luo et al. [7] discovered 164 differentially expressed miRNAs (DEMs) between the three skin colors (black, red and white) of Koi carp (*Cyprinus carpio* L.) such as miR-196a, miR-202 and miR-206. Hao et al. [8] identified 60 DEMs between the red-colored skin and black-colored of leopard coral grouper (*Plectropomus leopardus*). Meanwhile, Yin et al. [9] investigated that miR-196a regulates the skin pigmentation of koi carp by targeting transcription factor *mitfa* (melanocyte inducing transcription factor a). Other research also found that miR-196 family played a critical role in eye formation [10], fin bud initiation and regeneration [11] during the early development stages. These many pieces of information point to a potential role for miRNAs in the regulation of fish skin color.

Fish skin and scales frequently exhibit fascinating color patterns, and these patterns play crucial roles in a variety of biological processes, such as mating selection, camouflage, thermoregulation, photoprotection, mimicry and warning off predators [12]. Fish skin color is determined by a complicated process involving a series of cellular, genetic, environmental, nutritional and physiological factors [13]. As of now, many genes associated with the melanogenesis, the wnt signaling pathway and the MAPK signaling pathway, such as *oca*2 (oculocutaneous albinism II), *mitf*, *map2k4* (Mitogen-Activated Protein Kinase Kinase 4), *α*-*msh* (*α*-melanocyte stimulating hormone) and *foxd*3 (forkhead box d3), have been identified and are thought to be involved in the regulation of skin color [5, 14, 15]. But undoubtedly, the majority of the gene resources involved in determining fish skin color patterns are still under study.

Crucian carp (*C. auratus*), one of the most well-liked freshwater species in China with highly economic values, has undergone extensive artificial and natural selections throughout its evolution, and was able to adapt to a variety of environments after being bred into numerous strains for commercial production [16, 17]. The golden-back crucian carp (GBCrC) are distributed in the Dong’s Rice Fish Duck System (DRFDS), one of the Globally Important Agricultural Heritage Systems (GIAHS) in the world, which is located in Qiandongnan Miao and Dong Autonomous Prefecture, Guizhou Province, China. Due to its golden scales and skin on the dorsum, this fish was easier to observe in the paddy field. It has tender meat, soft bones and spines, and its appetizing soup is devoid of muck and fishy flavor. All of these make it worthy of scientific and reasonable breeding and exploitation. In our previous study, we investigated and examined its external morphological traits, distribution, paddy field selection, reproductive mode, genetic diversity and origin. The results showed that GBCrC has a gynogenesis reproductive mode and that the golden coloration of scales and skin is a mutation of native *C. auratus* that live under special conditions of altitude and annual temperature accumulation, and that this golden color does not disappear under other environmental conditions or through aquaculture methods [18]. Meanwhile, we also identified mRNAs and proteins that are closely associated with two different skin colors (golden and greenish grey) (data not yet published). However, there is still a gap in our knowledge of the genetics in the two skin differentiation types as well as the molecular regulatory mechanisms, which calls for further research employing advanced techniques.

Herein, we further identified skin color related miRNAs using the RNA-Seq approach and screened the DEMs between golden and greenish grey skin, and initially investigated how miR-196d contributed to the skin color differentiation and pigmentation in GBCrC. The findings will provide fundamental knowledge for understanding the genetic mechanisms underlying variation in color traits and may enable the exploitation of gene regulation in skin color determination.

## Results

### Overview miRNA expression signature in crucian carp skin

**Fig. 1:**
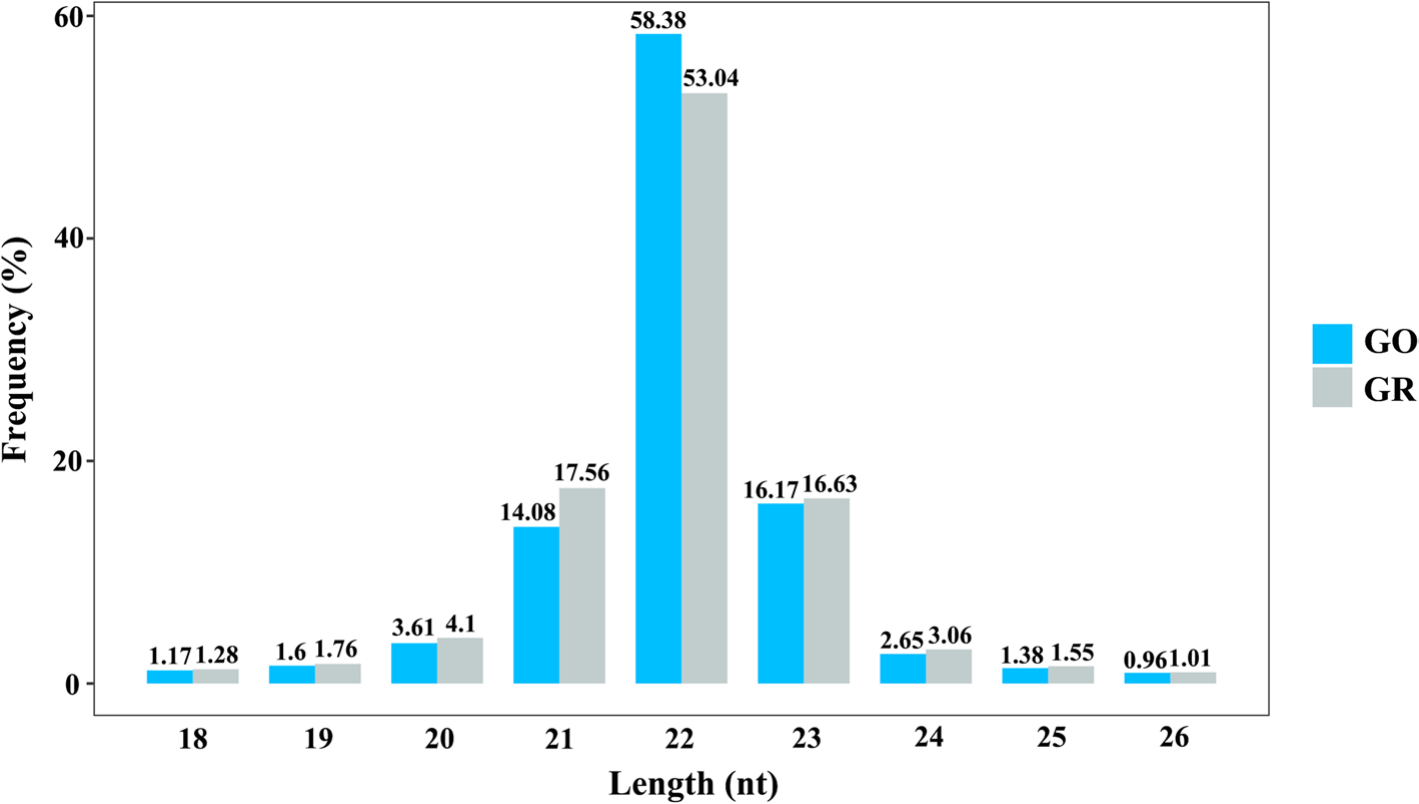
Length distribution of small RNAs in *C.auratus*

To identify miRNAs expressed in GBCrC skin, GO and GR skin small RNA libraries were analyzed by deep sequencing. A total of 11,555,738 and 10,941,036 raw reads were yielded from the two libraries, respectively (Table s1). After filtering out adaptor sequences, low quality reads, insert-null reads, and reads < 18 nt, the reads were further matched with databases for Repeat-associated RNA, GenBank, and Rfam (e.g., rRNA, tRNA, snRNA and snoRNA). Finally, 5,133,894 and 4,002,749 valid reads were retrieved for analysis (Table s1). Of these, 91.28% and 90.29% of the small RNA sequences obtained were 21–24 nt in size, which is the typical size range for Dicer-derived products (Fig. 1).

To further identify the conserved miRNAs and predicted novel miRNAs in the skin of GBCrC, blast search with an E-value cutoff of 10 was used to search for the remnant small RNAs with predicted hairpin structures against the miRbase 22.0. Following alignment and subsequent sequence analysis, 679 miRNAs were discovered to be identical to the mature miRNAs (known), 505 conservative miRNAs were found to match known precursor miRNAs of particular species and 254 miRNAs were projected to be novel miRNAs with hairpin structure (Table s2). Additionally, we counted 15 miRNAs (five highly expressed in both the golden skin and the greenish grey skin, five highly expressed in the golden skin only and five highly expressed in the greenish grey skin only) to better understand the miRNAs that are highly expressed in the skin of GBCrC (Table 1).

**Table 1:** The most abundant known miRNAs identified in *C.auratus* GR and GO samples

miRNA	Sequence	GR Count	GO Count	Expression
Oni-miR-199a	ACAGTAGTCTGCACATTGGTTA	103,814	107,267	Both highly
Oni-let-7a	TGAGGTAGTAGGTTGTATAGTT	71,880	69,635	Both highly
Dre-miR-125c-5p	TCCCTGAGACCCTAACTCGTGA	79,535	74,119	Both highly
Oni-miR-214	ACAGCAGGCACAGACAGGCAGT	68,102	64,174	Both highly
Ccr-miR-26a	TTCAAGTAATCCAGGATAGGCT	64,658	62,229	Both highly
Oni-miR-1	TGGAATGTAAAGAAGTATGTAT	220,886	434,073	Highly in GO
Ccr-miR-206	TGGAATGTAAGGAAGTGTGTGG	135,326	412,057	Highly in GO
Dre-miR-99	AACCCGTAGATCCGATCTTGTGA	189,032	204,614	Highly in GO
Ssa-miR-10b-5p	TACCCTGTAGAACCGAATTTGT	122,710	162,528	Highly in GO
Oni-miR-100	AACCCGTAGATCCGAACTTGTG	104,027	135,790	Highly in GO
Ccr-miR-22a	AAGCTGCCAGCTGAAGAACTGT	148,259	95,362	Highly in GR
Ccr-miR-21	TAGCTTATCAGACTGGTGTTGG	119,766	96,360	Highly in GR
oni-miR-101a	TACAGTACTGTGATAACTGAAG	94,066	78,489	Highly in GR
gmo-miR-126-3p	TCGTACCGTGAGTAATAATGCA	87,704	64,501	Highly in GR
Oni-miR-204a	TTCCCTTTGTCATCCTATGCCT	60,423	32,996	Highly in GR

### Differential expression of miRNAs in different skin

**Fig. 2:**
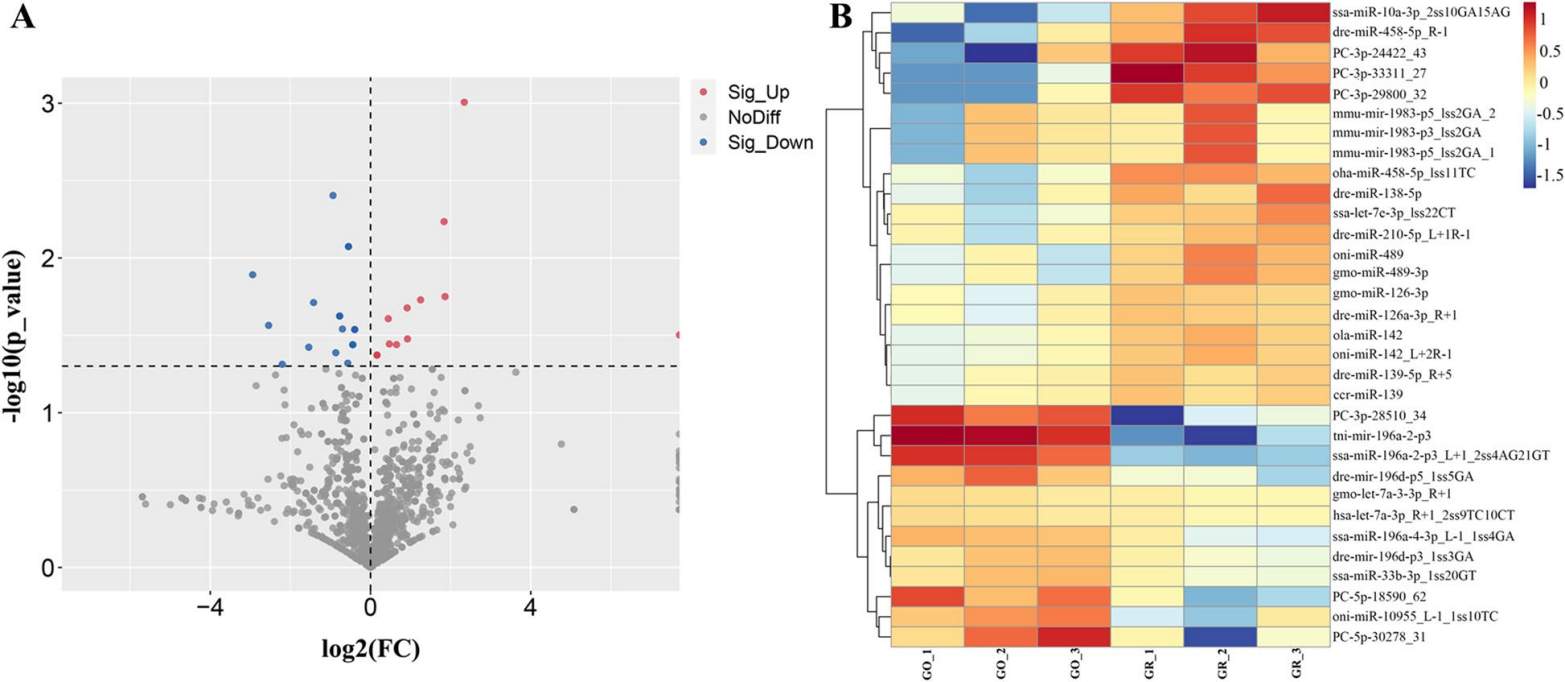
Differentially expressed miRNAs (DEMs) between the golden and greenish grey skin of *C.auratus*. **A** Volcano plot of DEMs between the GO and GR groups, gray, red, and blue dots represent non-significant, up-regulated, and down-regulated miRNAs, respectively; (**B**) A heatmap of all DEMs shows the expression levels, which ranged from low to high, from blue to red

**Fig. 3:**
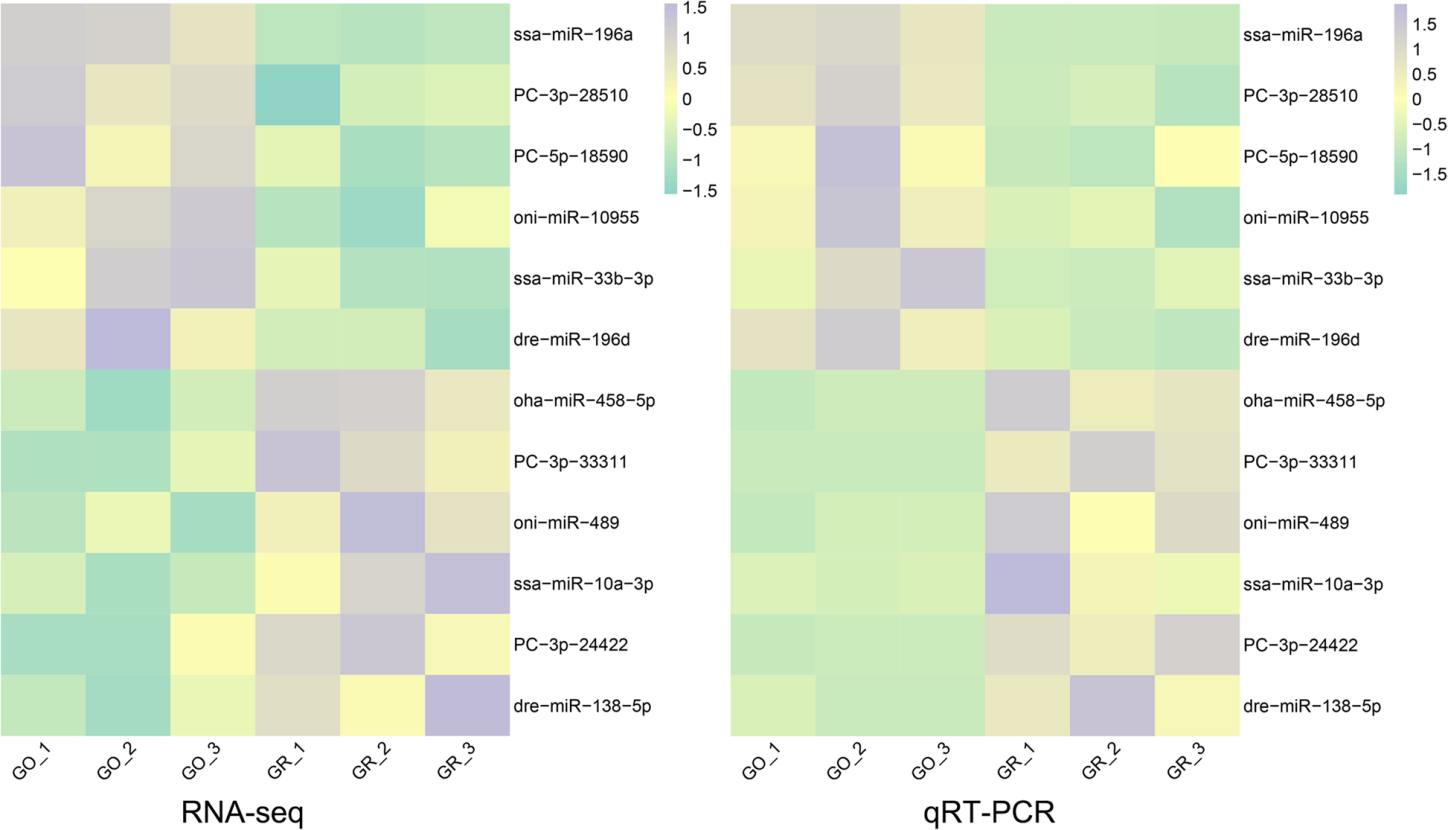
Heatmap analysis for RNA sequencing transcripts per million (TPM) and qRT-PCR relative expression of 12 differentially expressed miRNAs

To gain insight into the function of miRNAs in pigmentation processes, it is essential to have precise information on their expression patterns. With the criteria of *p*-value ≤ 0.05, we identified 32 significant DEMs in GO (including 12 up-regulated and 20 down-regulated miRNAs) compared with GR samples (Table s3). By drawing a volcano plot (Fig. 2A), we were able to better understand the general distribution of DEMs and discovered that certain miRNAs with point overlap due to their relatively close *p* values (e.g., gmo-miR-126-3p and dre-miR-126a-3p, gmo-let-7a-3-3p and hsa-let-7a-3p, etc.). Six novel predicted miRNAs were discovered among these miRNAs, three of which were strongly up-regulated in golden skin (PC-3p-28150, dre-mir-196d-p3 and ssa-miR-196a), and three of which were highly expressed in greenish grey skin (PC-3p-33311, PC-3p-29800, and PC-3p-24422) (Fig. 2B). Following that, we used qRT-PCR to confirm the expression patterns in 12 DEMs (including 6 down-regulated miRNAs and 6 up-regulated miRNAs). Our qRT-PCR expression patterns matched the results of RNA-seq analysis, indicating that the validity of the RNA-seq data (Fig. 3 and Table s4). For example, based on the small RNA sequencing results, the expression level of dre-miR-196d in GO was almost 1.894 times higher than in GR, compared with a 3.168-fold difference in the qRT-PCR results.

### Target genes prediction and enrichment analysis

**Fig. 4:**
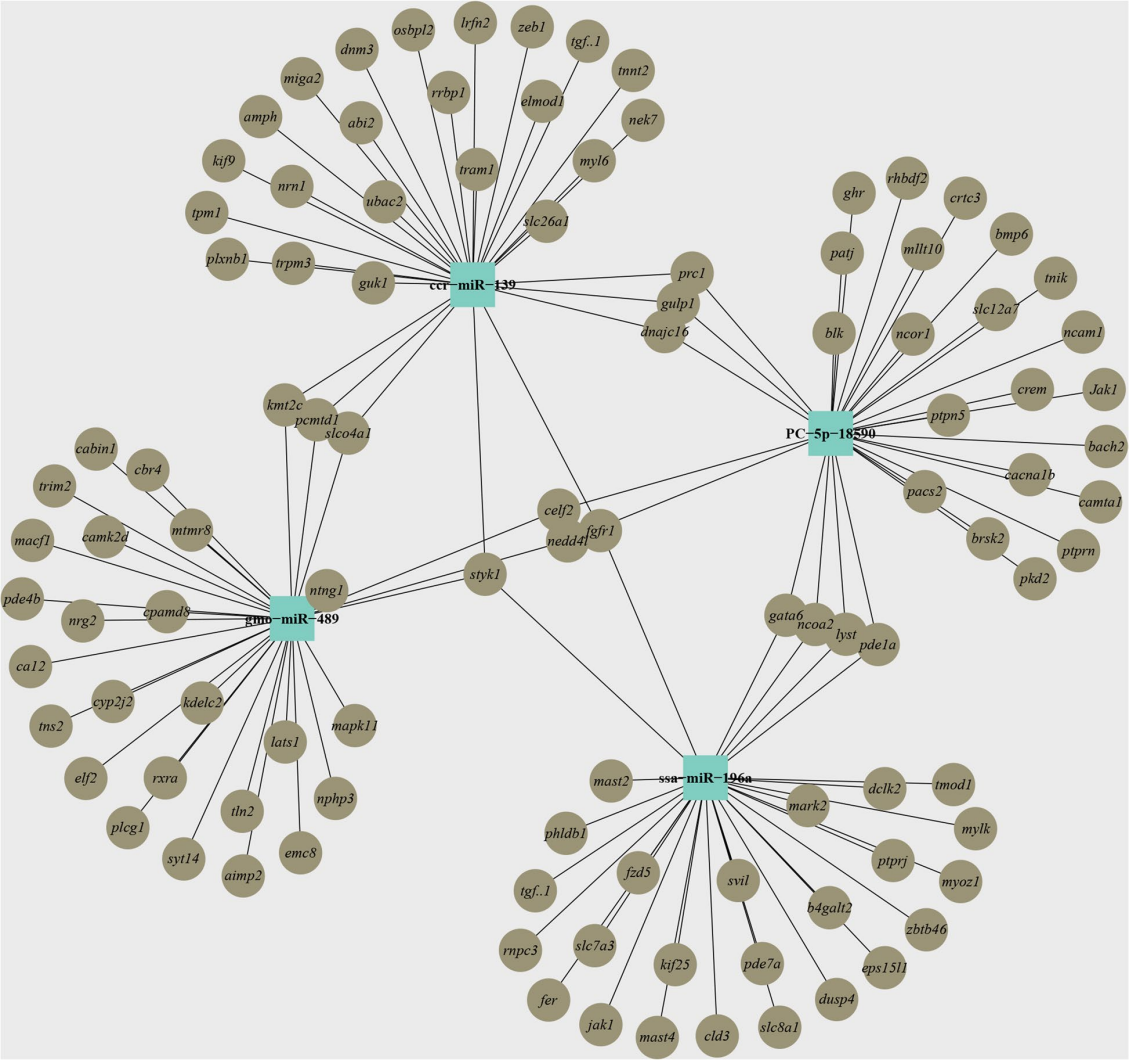
A putative network graph of interactions between miRNAs (cyan squares) and their target genes (brown circles)

Target prediction analysis was performed using the miRanda and TargetScan algorithms to further explore the potential functions of DEMs in various colored skin. The outcomes demonstrated that 46,011 transcripts and 23,577 genes could be targeted by 32 miRNAs with considerably differential expression. The miRNAs might target one or more transcripts, and some target transcripts might be regulated by multiple miRNAs (Table s5). For instance, ssa-miR-196a, ccr-miR-139, PC-5p-18590 and gmo-miR-489 can target 4402, 5443, 8420 and 5593 transcripts, respectively. Among these, genes like *styk*1 (serine/threonine/tyrosine kinase 1), *fgfr*1 (fibroblast growth factor receptor 1), *celf*2 (CUGBP elav-like family member 2) and *slco*4*a*1 (solute carrier organic anion transporter family member 4A1) can be regulated by numerous miRNAs simultaneously, indicating a complicated regulatory network of these DEMs in different skin differentiation and pigmentation (Fig. 4).

**Fig. 5:**
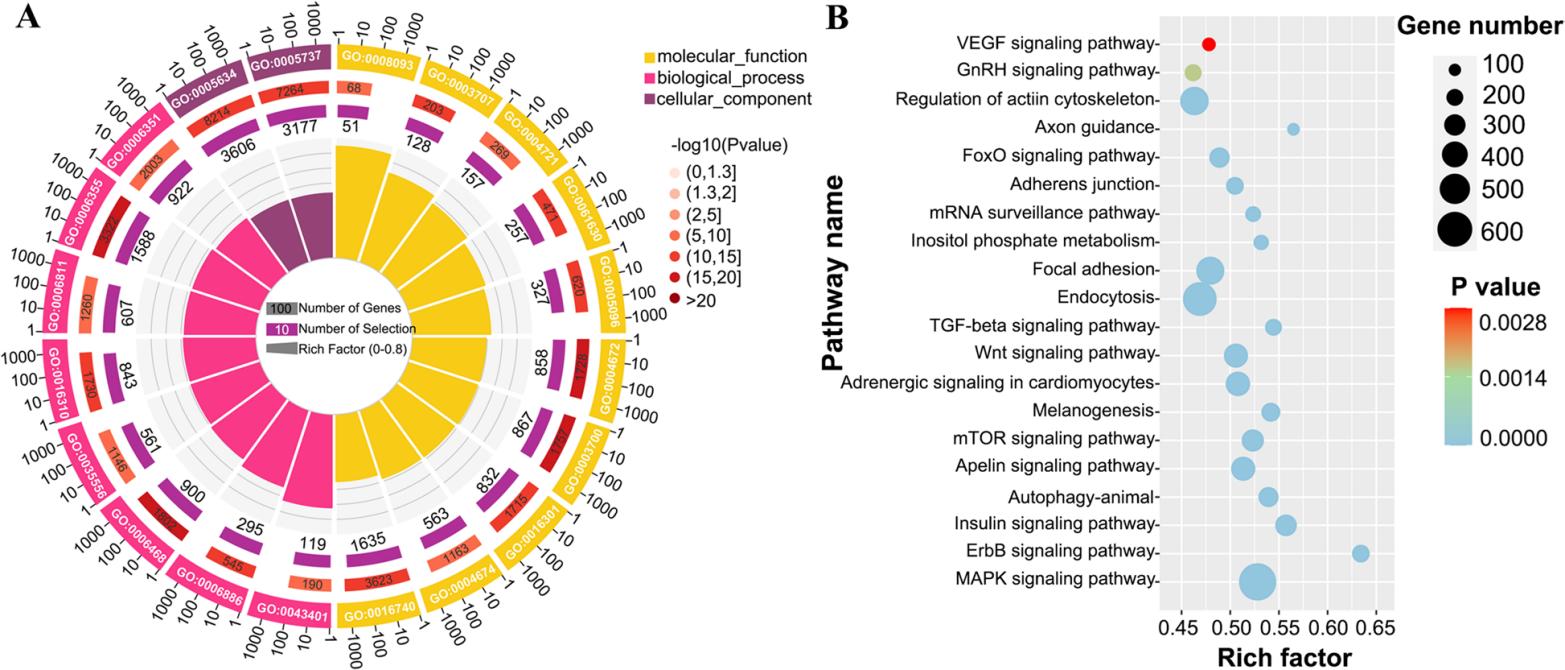
GO and KEGG analysis of the targets of the total differentially expressed miRNAs. **A** The top GO biology process terms, the circles from the outside to the inside indicate the GO ID, the number of significantly different genes and the number of significantly different genes annotated as specific GO, respectively. **B** the top KEGG pathways

Based on the functional annotation of the target transcripts, enrichment analysis (GO and KEGG) was carried out, and a number of biological processes and pathways were noticeably identified (Table s6). Intriguingly, we discovered that miRNAs can be markedly enriched to biological processes such as protein phosphorylation (GO:0,006,468), regulation of transcription, DNA-templated (GO:0,006,355) and ion transport (GO:0,006,811), molecular function such as transferase activity (GO:0,016,740), protein kinase activity (GO:0,004,672) and metal ion binding (GO:0,046,872), and cellular component such as nucleus (GO:0,005,634) and cytoplasm (GO:0,005,737) (Fig. 5A). Additionally, some KEGG pathways, such as Wnt signaling network, MAPK signaling pathway, melanogenesis and adrenergic signaling in cardiomyocytes, appear to be involved in the color differentiation and pigmentation of the *C.auratus* skin (Fig. 5B).

### Comprehensive regulation of miRNAs on their target expression

**Fig. 6:**
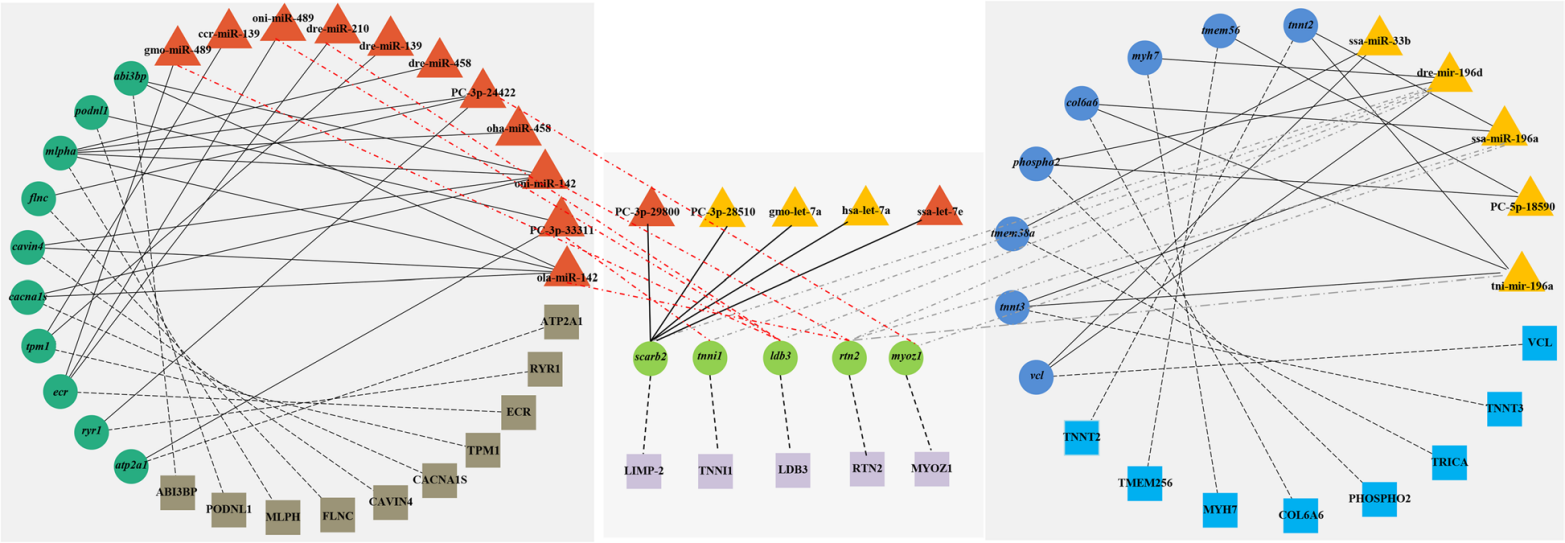
Analysis of the interaction between miRNA, mRNA and protein. The down-regulated and up-regulated miRNAs are shown as orange and yellow triangles, the corresponding mRNAs and proteins are shown as green and light blue circles, brown and blue squares, and the mRNAs and proteins of multiple relationship pairs are shown as light green circles and purple squares

In order to search for the targets of DEMs and to understand the impacts of post-transcriptional regulation of miRNAs on gene expression in different skin color pigmentations, we further combined transcriptomic and proteomic data to analysis the molecular networks of miRNA targets. For miRNA-mRNA-protein association analysis, data that met the criteria were screened. Then, we determined 52 groups of reciprocal interaction networks matching the criteria and separated them into two main groups based on the 23 up-regulation and 29 down-regulation of miRNAs (Table s7). Meanwhile, we also discovered some features of miRNA-regulated expression, such as: 1) there were six expression patterns of miRNAs-mRNAs-proteins (down-down-down, down-down-up, down-up-down, down-up-up, up-down-down and up-up-up), of which down-up-up and up-down-down regulation ways were relatively high; 2) one miRNA could target and regulate multiple mRNAs simultaneously, such as ssa-miR-196a could target *tnnt*2 (*troponin T*, *cardiac muscle isoforms-like*), *rtn*2 (*reticulon-2-like*), *col*6*a*6 (*collagen alpha*-6 (VI) *chain-like*) and *myoz*1 (*myozenin*-1-*like*) (Fig. 6); 3) one mRNA or protein could be regulated by numerous miRNAs, such as the *scarb*2 (*lysosome membrane protein* 2-*like*) gene and LIMP-2 (lysosomal integral membrane protein type 2) protein are simultaneously regulated by PC-3p-29800, PC-3p-28510, gmo-let-7a, has-let-7a and ssa-let-7e (Fig. 6). As a result, miRNAs exhibited a more complicated regulation of their target expression, indicated by both repression and activation at the transcriptional and translational levels.

Then, we conducted GO annotation and KEGG enrichment analysis on these highly differentially expressed miRNA-mRNA-protein interactions (Table s7). Several significant biology processes (e.g., sarcomere organization, ion transport, and calcium ion transport), cellular component (e.g., membrane, integral component of membrane, and cytoplasm), molecular function (e.g., actin binding, actin filament binding, and protein binding) (Fig. s1) and pathways (e.g., MAPK signaling pathway, focal adhesion, and adrenergic signaling in cardiomyocytes) (Fig. s2) were enriched.

### The role of miR-196d involved in the skin pigmentation

**Fig. 7:**
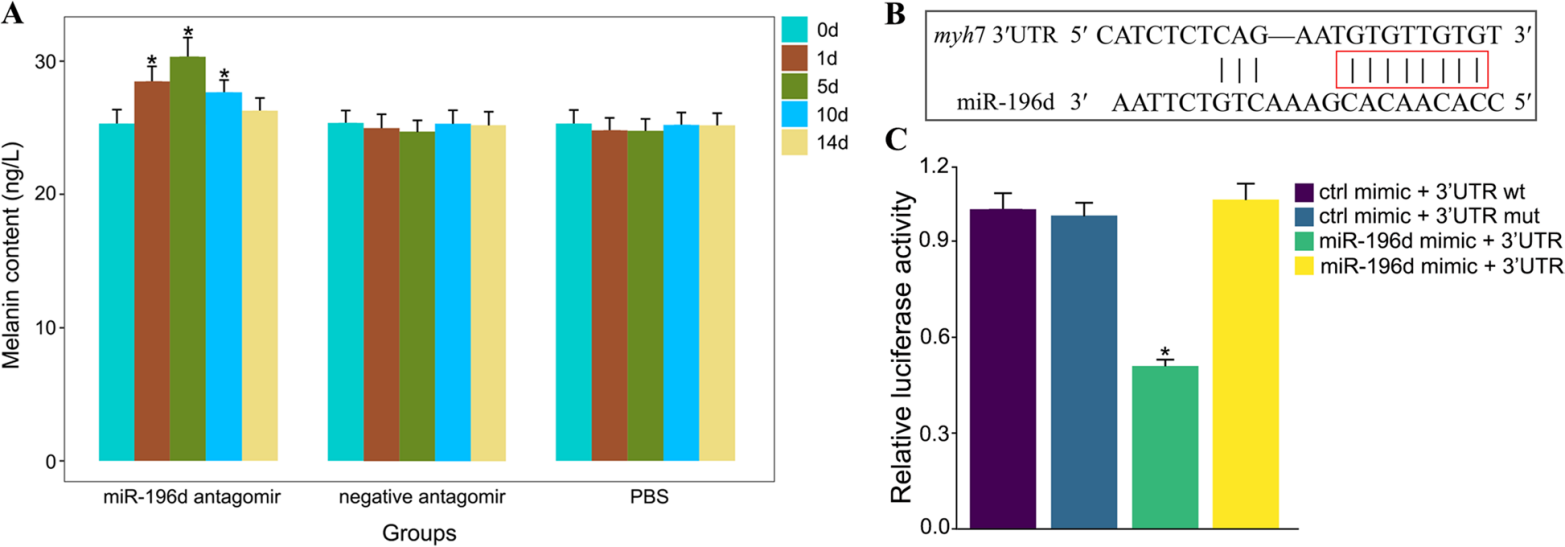
**A** The pattern of changes in melanin content at different times in various treatment groups. **B** Schematic diagram of predicted miR-196d binding sites in the 3′-UTR of *myh*7. **C** Dual luciferase reporter assays system was used to analysis binding between miR-196d and *myh*7. The treatment of ctrl mimic + 3′UTR wt was taken as the control group. Asterisk (*) indicates a significant difference compared with the control group (*p* < 0.05)

In our previous studies, we conducted in vivo investigations on miRNA function using the miRNA antagomir approach. Here, we also discovered that miR-196d expression in fish could be efficiently suppressed by its antagomir at a level of 40 mg/kg through the tail vein injection, but not by the mismatched antagomir (Fig. s3). Then, we used miR-196d antagomir (positive control), negative antagomir (negative control), and PBS (blank control) to treat wild-type GBCrC. We discovered that the GBCrC skin had a higher melanin content after miR-196d antagomir injection relative to its negative controls (Fig. 7A). In addition, we also examined the tissue-specific expression profiles of miR-196d, and discovered that it was strongly expressed in the gonad and brain, followed by golden skin, heart and fin, but not in the liver, intestine or gill (Fig. s4). These findings suggested that miR-196d expression levels might have an impact on fish skin pigmentation.

To elucidate the role of miR-196d in the skin pigmentation process, we further explored it in conjunction with data from sRNA sequencing and identified the *myh*7 as a potential target gene in the adrenergic signaling pathway. The alignment of miR-196d with *myh*7 3′-UTR is illustrated in Fig. 7B based on the sequence complementary. Additionally, we used two luciferase reporters, which were the wild-type 3′-UTR and the mutant 3′-UTR of *myh*7. These luciferase reporters were co-transfected into HEK293T cells together with a miR-196d mimic. To regulate the non-specific effects of expression, a scrambled miRNA mimic that has no similarity to the *C.auratus* genome was utilized. The findings showed that the miR-196d mimic dramatically reduced the luciferase activity of the wild-type *myh*7 3′-UTR but had no effect on the luciferase activity of the mutant 3′-UTR, indicating that miR-196d directly inhibited *myh*7 expression by binding to its 3′-UTR regions (Fig. 7C).

**Fig. 8:**
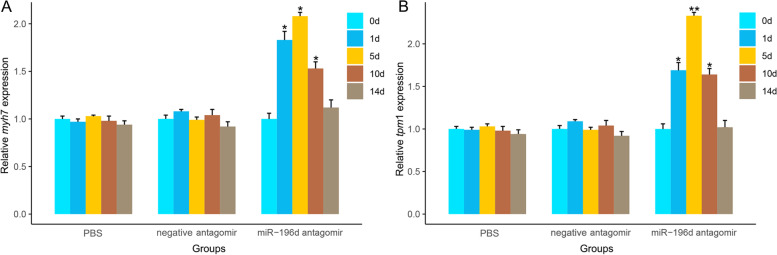
Effect of miR-196d silencing on expression of *myh*7 (**A**) and *tpm*1 (**B**) genes at different time points, *β-actin* expression was detected as the internal control. * *p* < 0.05, ** *p* < 0.01

Meanwhile, we further detected the expression levels of *myh*7 and *tpm*1 (*tropomyosin alpha*-1 *chain isoform X*16) genes, which are related to the adrenergic signaling pathway. The findings indicated that the expression levels in the miR-196d antagomir injection group were all significantly higher than those in the controls within 1 day, 5 days, and 10 days (*p* < 0.05), suggesting that miR-196d silencing can affect the expression of genes involved in the adrenergic signaling pathway and regulate the production of melanin (Fig. 8).

## Discussion

In fish, the formation of body color is caused by the light absorption of biological pigments or by the interference of chromatophores with light-reflecting substances, whereas color patterns are produced by the combination and distribution of different pigment cell types [19, 20]. In a previous study, we identified the distinct phenotype of GBCrC and suggested that two golden regions (one is from the 3^rd^ squama on the dorsum to the origin of dorsal fin, and another is from the base of 6^th^-10^th^ fin ray of the dorsal fin to the base of caudal fin) might result from special altitudes and annual accumulated temperatures [18]. In order to better understand the molecular roles underlying the differentiation and pigmentation of different skin colors, the current study sorted the DEMs that were closely related to them and conducted a preliminary study on miR-196d function, with the aim of laying the theoretical groundwork for the subsequent deeper investigation of the regulatory mechanisms.

MiRNAs play pivotal roles in various biological processes by promoting mRNA degradation or inhibiting mRNA translation, and increasing evidences suggest that their dysregulations have an impact on the skin differentiation and pigmentation [21, 22]. Herein, our sequencing results revealed a similar read length distribution of 21–24 nt, which is comparable in size to typical Dicer-derived products [23]. Most of the identified miRNAs including let-7a, miR-199a, miR-214, miR-1, etc., were also shown to be abundantly expressed in other species, such as koi carp [7], rainbow trout (*Oncorhynchus mykiss*) [24] and Japanese flounder (*Paralichthys olivaceus*) [25]. Similarly, miR-138-5p and let-7a were also predicted to be highly expressed in the miRNA sequencing investigation of the diverse body colors of red tilapia [6] and Manila clam (*Ruditapes philippinarum*) [26]. Studies have shown that miR-33b, which specifically targets hypoxia inducible factor (*hif*)-1*α*, can reduce malignant melanoma cell proliferation and glycolysis [27]. Meanwhile, it has been demonstrated that miRNA-139 could bind to its target gene *igf*1*r* (insulin-like growth factor receptor type 1) to regulate the PI3K/AKT signaling pathway, which in turn controls the proliferation and spread of malignant melanoma cells [28]. As a result, it is likely that our subsequent research will concentrate on how these miRNAs affect the skin-color regulation of *C. auratus*.

MiRNAs paly a variety of biological functions, including targeting transcripts and suppressing post-transcriptional gene expression [1]. By target prediction and examination of the correlation between their expression levels, we further investigated the potential relationship between miRNAs and genes. The results showed the complexity of the miRNA-mRNA network and the potential for a single miRNA to target multiple genes and for numerous miRNAs to target the same gene. Similar findings in other research have suggested that particular miRNAs might be involved in the gene regulation of numerous physiologically active processes, and this observation supports the idea of functional redundancy among miRNAs [25, 29]. MiRNAs in various cell types form a comprehensive, multi-layered network system through interactions with signaling pathways and regulatory elements [30].

Additionally, we performed enrichment analysis on the targeted genes to better understand the molecular function. Among these, GO function analysis is frequently used to explain the molecular function, cellular component and associated biological process. We discovered that several GO terms, including protein phosphorylation (GO:0,006,468), transcription regulation and DNA-templated (GO:0,006,355), nucleus (GO: 0,005,634) and cytoplasm (GO: 0,005,737), can be significantly enriched in biological processes (Fig. 5A), suggesting that the various biological functions underlying the color variations in *C.auratus* skin. Furthermore, the transcription factor binding (GO: 0,008,134), which is crucial to controlling the transcription of proteins in cells, primarily reflects the enrichment difference between two skin colors. The MAPK, PI3K/Akt, Wnt/*β*-catenin signaling pathway and melanogenesis pathway are the important regulatory pathways that promote or inhibit the melanin synthesis [31]. KEGG pathway analysis showed that several different pathways for DEMs to target genes were classified, including the adrenergic signaling in cardiomyocyte (ko04261), MAPK signaling pathway (ko04010), wnt signaling pathway (ko04310) and melanogenesis (ko04916), which might be involved in pigment formation and regulation (Table s6). The MAPK signaling pathway, which involved 630 genes, was the main one among them. Previous research has shown that members of the MAPK family proteins, including p38, ERK and JNK, are essential for melanogenesis and p38 also activates MITF, which can increase the expression of melanogenic enzymes [32]. While through suppressing MITF, the ERK and/or JNK/SAPK pathways cause the down-regulation of melanin synthesis [33]. Additionally, the wnt signaling pathway has been shown to be involved in regulating pigment cell differentiation in zebrafish (*Danio rerio*) [34], and the relationship between Wnt/*β*-catenin signaling and MITF has been reported as a key feature of melanocyte development and subsequent pigmentation [35]. It is therefore obvious that different genes in each of these pathways may be involved in regulating skin production in various color phenotypes, but the precise mechanism of action requires further research.

In general, the type of complementarity between miRNAs and their target genes dictates the regulatory interaction, which typically involves two post-transcriptional regulatory mechanisms, mRNA cleavage or translational repression, both of which negatively influence the expression of target genes [36]. However, the mRNA expression profile and protein level of the same genes can be different due to various factors in regulatory mechanisms (e.g., RNA secondary structure, the effects of regulatory proteins, and codon bias) [37]. In our study, we found there were six expression patterns of miRNAs-mRNAs-proteins (down-down-down, down-down-up, down-up-down, down-up-up, up-down-down, and up-up-up). What is more, the expression direction of some miRNAs, their target genes and proteins (e.g., miR-196d-*myh*7-MYH7, let-7e-*scarb*2-LIMP-2 and PC-3p-33311-*podnl*1 (podocan-like 1)-PODNL1) was the same. This might be under two potential mechanisms: 1) counter-regulation of different post-transcriptional mechanisms and 2) the down-regulation of protein production being offset by a feedback mechanism [38]. On the other hand, a small number of miRNAs can regulate protein expression by pairing with proteins to the opposite-expression direction (e.g., miR-142-*mlpha* (melanophilin *a*)-MLPH, miR-458-5p-*mlpha*-MLPH and PC-3p-24422-*flnc* (filamin C)-FLNC). This is in line with previous study that most animal miRNAs do not precisely complement their targets, leading to their primary mode of action being translational suppression rather than mRNA breakage and destruction [39]. According to our research, it is critical to take into account both aspects of miRNA regulation, including translational repression that results in activation during skin color differentiation and pigmentation.

Pigment cells typically have small muscle fibers and nerve endings distributed on the membrane. The primary cause of the variation in body color in fish is due to these cells changing their morphology when muscle fibers contract, causing the pigment particles to shift under the influence of kinesin, and thus scattering or accumulating in different areas of the body surface [40]. It has been demonstrated that melanocytes had *β*-adrenergic receptors, and that the adrenaline (AD) was responsible for the diffusion of melanin granules to darken the body color of animals, whereas noradrenaline (NE) was in charge of the aggregation of pigments within melanophores to lighten animals’ bodies, both belonged to the category of neuromodulation of body color changes [41]. Adrenergic receptor (AR) belongs to G protein-coupled receptors, which are receptor proteins for the action of catecholamines and are divided into four subtypes, including *α*1, *α*2, *β*1 and *β*2 [42]. Among them, *α*2-AR agonist regulates the production of pigment in melanocytes and erythrophores, includes the aggregation of melanin granules through mediating NE and affects the melanocyte apoptosis; *β*2-AR stimulates adenylate cyclase (cAMP), a key step comparable to the regulation of melanogenesis pathway, and will mediate AD to inhibit melanocyte aggregation [43]. In this study, we discovered that some miRNAs with differential expression (such as miR-196d, miR-139 and PC-3p-24422) were significantly enriched to key members of the adrenergic signaling pathway, including *tpm*1, *myoz*1, *myh*7 and *act*3 (actin related gene 3), suggesting that these miRNAs may be involved in regulating the development of various pigment cells. Therefore, we further explored how miR-196d involved in regulating the differentiation and pigmentation process of skin color by targeting the *myh*7 gene.

**Fig. 9:**
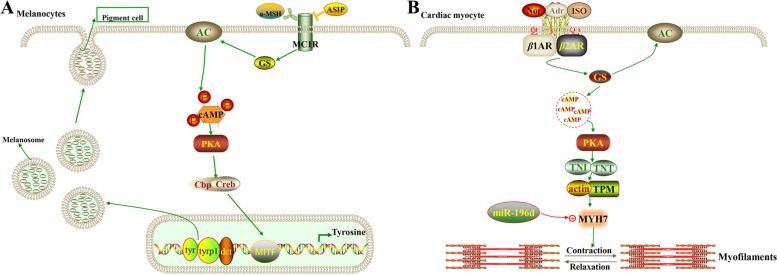
**A** The possible regulatory pathway of melanogenesis activation. **B** The biological effects of miR-196d in the determination of skin color in the adrenergic signaling in cardiomyocyte pathway

Antagomirs are single-stranded RNA molecules (21–23 nt) conjugated to cholesterol that are complementary to a particular miRNA target and contain either a base change or a mispairing at the Ago2 cleavage site to prevent Ago2 cleavage [44]. In our previous studies, we employed a miRNA antagomir technique to carry out in vivo miRNA loss-of-function assays [8, 45]. Herein, we also found that miR-196d expression could be effectively inhibited in vivo by its equivalent antagomir but not the mismatched miRNA. The *α*-MSH peptide participates in the melanogenesis process by binding to *mc*1*r*, raising intracellular cAMP levels, promoting adenylyl cyclase, and activating protein kinase A, which in turn activates tyrosinase to create melanin (Fig. 9A) [46]. Levodopa, an intermediate product of melanin synthesis, is another metabolic pathway that forms catecholamines, which are converted to dopamine, NE and AD by the action of the corresponding transferase enzymes, which can phosphorylate tyrosine and thus regulate tyrosinase activity through the AC/cAMP kinase system and affect melanin content [41]. It has been demonstrated that in the adrenergic signaling pathway, AD binded to *β*1-AR or *β*2-AR receptors to activate Gs proteins, which influenced the concentration of the second messenger cAMP by activating adenylate cyclase (AC), and ultimately affected the expression of genes like *act*3, *tpm*1 and *myh*7 through activating the protein kinase A system (PKA) and Ca^2+^ signaling pathway [47]. In the present study, we found a binding site of miR-196d in the 3′-UTR regions of *myh*7, and characterized their effects on *myh*7 using a reporter assay. Meanwhile, we also discovered that the miR-196d antagonist injection group had higher levels of *myh*7 and *tpm*1 gene expression than control groups, implying that miRNA-196d, which targeted the *myh*7 gene in the *β*2AR-GS-AC-cAMP-PKA signal transduction pathway, may indirectly regulate the synthesis of melanin in crucian carp (Fig. 9B).

In summary, the differentiation and pigmentation of *C.auratus* skin is a complex event involving several genes and pathways that interact cross-talk. We provided evidence that miRNAs emerged as crucial roles in these processes, and comprehending the regulatory mechanism at the post-transcription level will offer fresh perspectives on the regulation of skin color development and formation.

## Conclusions

Our study provides basic information on miRNAs expression in GBCrC, including knowledge of known and potential novel miRNAs, as well as understanding of DEMs related to different skin colors and their associated target genes. We built an interaction module of mRNAs, proteins and miRNAs based on 10 up-regulated and 13 down-regulated miRNAs in golden skin, and found that miR-196d, which targeted the *myh*7 gene may be indirectly implicated in regulating skin melanocyte synthesis and motility. The findings would provide to a better understanding of the complex molecular regulation of *C.auratus* body color polymorphism.

## Methods

### Sample collection

One hundred eight-month-old female GBCrC (average weight: 40 ± 3 g) were collected from paddy fields in Pingzheng village, which is a part of the DRFDS and is located at 25°38′N-108°39′E in Congjiang County, Qiandongnan Miao and Dong Autonomous Prefecture, Guizhou, China. They were kept in a water circulation system in 200-L tanks under 12-h light/dark photoperiod at 24 ± 1 °C. Then, three fish were tranquilized in 15 ~ 20 mg/L MS-222 buffered to pH 7.0 ~ 7.5, and golden skin (GO) and greenish grey skin (GR) were collected for small RNA library construction. Meanwhile, the other three fish samples (GO, GR, gonad, gill, liver, muscle, blood, heart, caudal fin, spleen, intestine, eye and brain) were collected for functional analysis. All samples were immediately snap-frozen in liquid nitrogen and stored at -80℃ until use.

### Small RNA library construction and sequencing analysis

RNA samples were obtained from different colored skin of GBCrC using TRIzol (Invitrogen, USA) according to the manufacturer’s protocol. The purity and quantity were analyzed by RNA Nano LabChip (Agilent, USA) and Bioanalyzer 2100 with RIN value > 7.0, then kept in -80℃ refrigerator. Small RNA libraries were constructed following the protocol of Illumina’s TruSeq Small RNA Sample Preparation Kits (San Diego, CA, USA). After that, single-end sequencing (50 bp) was run followed the manufacturer’s instructions on an Illumina Hiseq 2500 at the LC-BIO (Hangzhou, China).

Raw reads were subjected to ACGT101-miR program (LC Sciences, Houston, USA) [48] to remove adapter dimers, low complexity, common RNA families (rRNA, tRNA, snRNA, snoRNA) and repeats. Subsequently, unique sequences (18 ~ 26 nucleotide) were mapped to the *C.auratus* genome (https://www.ncbi.nlm.nih.gov/genome/?term=goldfish) by blast with a tolerance of one mismatch. The mapped sequences were separated into two categories: known miRNAs, which are found at the hairpin arms, and novel miRNAs, which are found at the arm that is opposite the annotated arm. After that, the remaining sequences were mapped to other fish species precursors in miRBase 22.0 by BLAST search, and the mapped pre-miRNAs were further blasted against the *C.auratus* genome to determine their genomic locations. To identify the novel predicted miRNAs, the unmapped sequences were blasted against the *C.auratus* genome database, and the hairpin RNA structures comprising sequences were predicated by RNAfold software (http://rna.tbi.univie.ac. at/cgi-bin/RNAfold.cgi).

### Differentially expressed miRNAs and target genes prediction

The methods used for data normalization were those outlined in a prior study [49]. miRNAs were regarded as differentially expressed based on normalized deep-sequencing levels in greenish grey and golden skin. The *p*-value was calculated by using Chi-square (X^2^) test and Fisher exact test. The significance level for each test was set at 0.05. Those miRNAs with *p*-value ≤ 0.05 were considered as DEMs.

Two computational target prediction algorithms, TargetScan [50] and Miranda [51], were utilized to locate miRNA binding sites in order to anticipate the genes that the most prevalent miRNAs will target. The overlaps were then determined using the combined data predicted by the two methods. Gene Ontology (GO; http://www.geneontology.org) biological process categories were used to identify functions strongly associated with the predicted target genes of miRNAs [52] and KEGG enrichment analysis was carried out using the pathway database (http://www.genome.jp/kegg/pathway.html) in a statistical test on each DEMs [53, 54], and the results were plotted using R program v3.0.1 (https://cran-archive.r-project.org/bin/windows/base/old/3.0.1/).

Based on the results of the previous transcriptome and proteome sequencing of golden back crucian carp with GO and GR samples (data not yet published), we performed a triple association analysis for differential mRNAs satisfying FC > 2 or FC < 0.5 with *p* < 0.05, differential proteins satisfying FC > 1.2 or FC < 0.833 with *p* < 0.05, and differential miRNAs satisfying* p* < 0.05. For analysis, Cytoscape v2.8.1 (https://cytoscape.org/download.html) plotted and networked the outcome files.

### Silencing miR-196d in vivo with the antagomir

The antagomirs employed are single-stranded RNA that consists of 21–23 nucleotides by chemically modified as follows: the miR-196d antagomir is CsCsCCACAACACGAAACUGUUAAsCsCsAs-Chol-3′. Additionally, we set up miRNA negative antagomir (CsCsACACACACUUCCUUACAUUsCsCsAs-Chol-3′) and phosphate-buffered saline (PBS) groups for the experiment’s control, respectively. The subscript “s” represents for phosphorothioate linkage and “Chol” stands for cholesterol connected via hydroxyproline in chemical modifications of miRNA antagomir, and all nucleotides are 2’-OMe-modified. Following that, 60 GBCrCs that were being temporarily raised in 200 l tanks were evenly divided into three groups that subjected to the tail vein injection of miR-196d antagomir, negative antagomir and PBS, respectively. Golden skin samples were taken at 0-, 1-, 5-, 10-, and 14-day after injection, then instantly frozen in liquid nitrogen and kept at -80 °C.

### Luciferase reporter assay

*Myh*7’s 3′-UTR was synthesized based on the crucian carp sequence (XM_026241629.1) and was then separately cloned into pSE3575 vector (Sunbio Medical Biotech Co., Ltd., China) by directional cloning. By altering the seed region of the predicted miR-196d site, the mutant *myh*7 3′-UTR reporters were produced. HEK293 cells, which do not express miR-196d, were seeded into 48-well plates at a density of 1 × 10^5^ cells per well the day before transfection. Then, using lipofectamine 2000 (Invitrogen, Carlsbad, CA, USA), 25 ng of the luciferase reporter vector carrying 50 nM miR-196d mimic, miR-196d wild-type (wt), or the 3′-UTR mutant was co-transfected with 5 ng of the *Renilla* luciferase control vector (pRL-TK, Promega, Madison, WI, USA) in 24-well plates. Using the Dual Luciferase Reporter Assay System (Promega, Madison, WI, USA), luciferase assays were performed in accordance with the manufacturer’s instructions at 48 h after transfection. A liquid scintillation counter was used to detect luciferase activity, the firefly luciferase activity was normalized to *Renilla* luciferase activity.

### Quantitative Real-Time PCR (qRT-PCR) analysis

Total RNA extraction procedures followed the same procedures as those above mentioned for small RNA libraries construction. The step-by-step process for qRT-PCR and reverse transcription is consistent with our earlier study (Dong et al., 2020). All the primers (Table s8) were designed using Primer Premier v5.0 and synthesized by Sangon Biotech., China. *U6* snRNA and *β*-actin were employed as the internal controls of the DEM and target genes, respectively, in three biological replications of each reaction. The relative expression values were determined based on the 2^-△△Ct^ method [55].

### Statistical analysis

The ELISA kit (Zhenke, Shanghai, China) was used to quantify the activities of melanin (ME), and the precise steps were the same as those described in the article by Wang et al. [6]. All results were presented as means ± standard deviation, and SPSS v21.0. (SPSS Inc., IL, USA) was used to analyze the data. By using a one-way ANOVA and post hoc Duncan’s multiple range tests, the sample points of various treatments were examined. *P* < 0.05 was deemed to be significant.

## Supplementary Information

**Additional file 1. Fig.s1.** Gene ontology (GO) enrichment analysis of mRNAs targeted by miRNAs that were significantly differentially expressed in the GO and GR groups.

**Additional file 2. Fig.s2.** Kyoto Encyclopedia of Genes and Genomes (KEGG) analysis of the targets of the total differentially expressed miRNAs (DEMs). Gene number, number of target genes in each pathway.

**Additional file 3. Fig.s3.** Effect of antagomir treatment on miR-196d expression. Fish was injected with miR-206 antagomir, miR-196d antagomir or left untreated, respectively. Asterisk (*) indicates a significant difference compared with the control group. Each sample was analyzed in triplicate.

**Additional file 4. Fig.s4.** Expression pattern of miR-196d in different tissues. Go, gonad; Br, brain; He, heart; Fi, fin; Gos, golden skin; Grs, greenish grey skin; Sp, spleen; Mu, muscle; In, intestine; Li, liver; Gi, gill; Ey, eye; Bl, blood. * p <0.05, ** p <0.01, *** p <0.001.

**Additional file 5: Table s1.** Distribution of sequenced reads from raw data to cleaned sequences. **Table s2.** Summary of known and predicted miRNAs in this study. **Table s3.** Differentially expressed miRNAs (DEMs) in different skin color of crucian carp. **Table s4.** The results of qRT-PCR and small RNA sequencing. **Table s5.** Results of target gene analysis of various miRNAs. **Table s6.** GO function and KEGG pathway enrichment analysis of differentially expressed miRNAs. **Table s7.** Information on the correspondence between differentially miRNAs, mRNAs, and proteins. **Table s8.** Primers for skin DEMs and reference miRNAs in crucian carp.

## Data Availability

The original contributions presented in the study are publicly available. All raw sRNA sequencing data have been deposited in the NCBI Gene Expression Omnibus (GEO) with the accession numbers GSE212497.

## Abbreviations

NCBI: National Center for Biotechnology Information
*Mitf*: Microphthalmia-associated transcription factor
*Mc*1*r*: Melanocortin receptor 1
*Msh*: Melanocyte-stimulating hormone
RISC: RNA-inducing silencing complex
RT-qPCR: Real-time quantitative reverse transcription polymerase chain reaction
FFRC: Freshwater Fisheries Research Center
DEMs: Differentially expressed miRNAs
GO: Gene ontology
KEGG: Kyoto Encyclopedia of Genes and Genomes
Wnt/β-catenin signaling: Wingless and INT-1 signaling transduction
MAPK: Mitogen-activated protein kinase
cAMP: Cyclic adenosine monophosphate
