# Supplementary figures and images for: microRNA regulation of skin pigmentation in golden-back mutant of crucian carp from a rice-fish integrated farming system
Source: BMC Genomics. 2023 Feb 10;24:70. doi: 10.1186/s12864-023-09168-w (PMC9912656; doi:10.1186/s12864-023-09168-w)

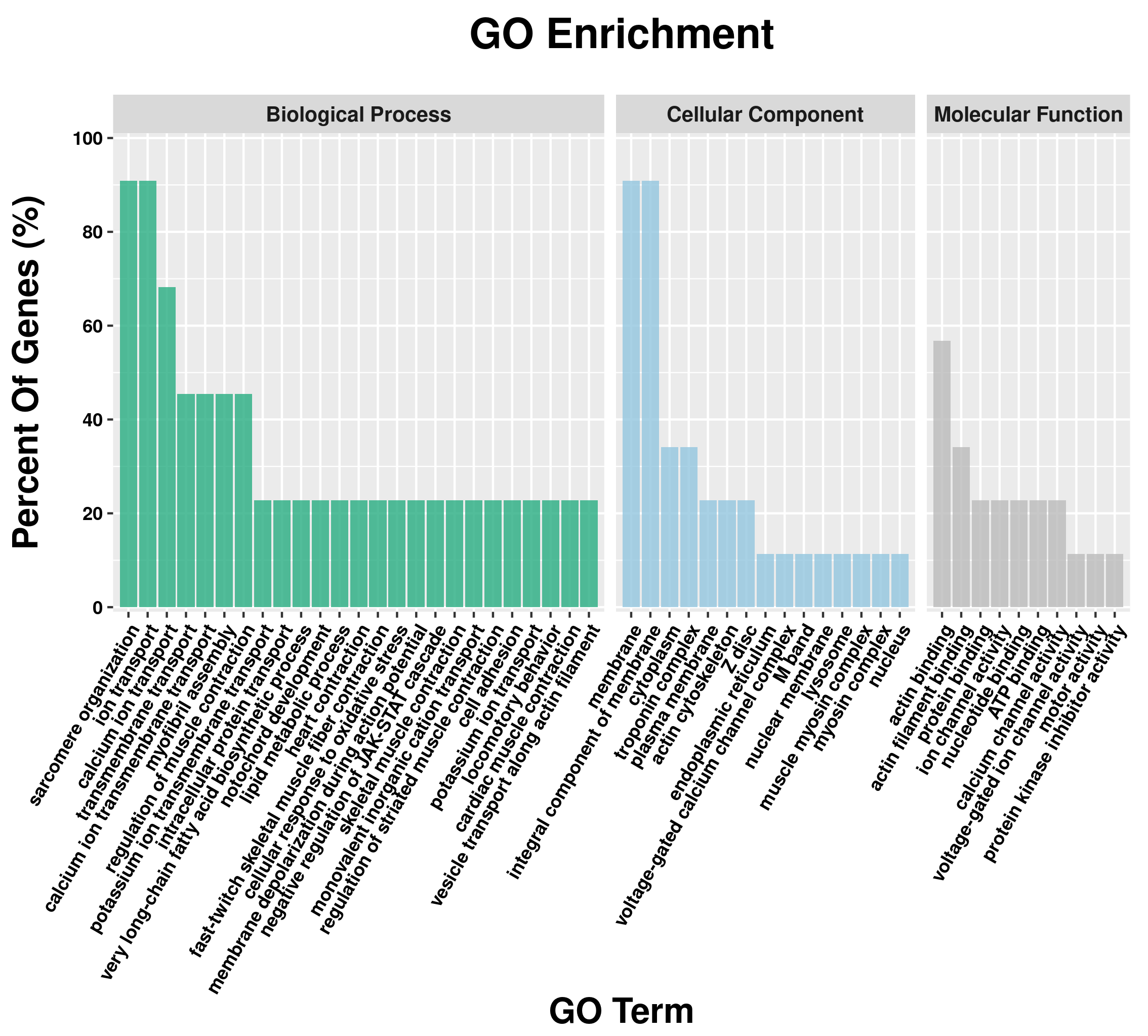

Supplement: Supplementary file 1 — Additional file 1. Fig.s1. Gene ontology (GO) enrichment analysis of mRNAs targeted by miRNAs that were significantly differentially expressed in the GO and GR groups. [file 12864_2023_9168_MOESM1_ESM.tiff]

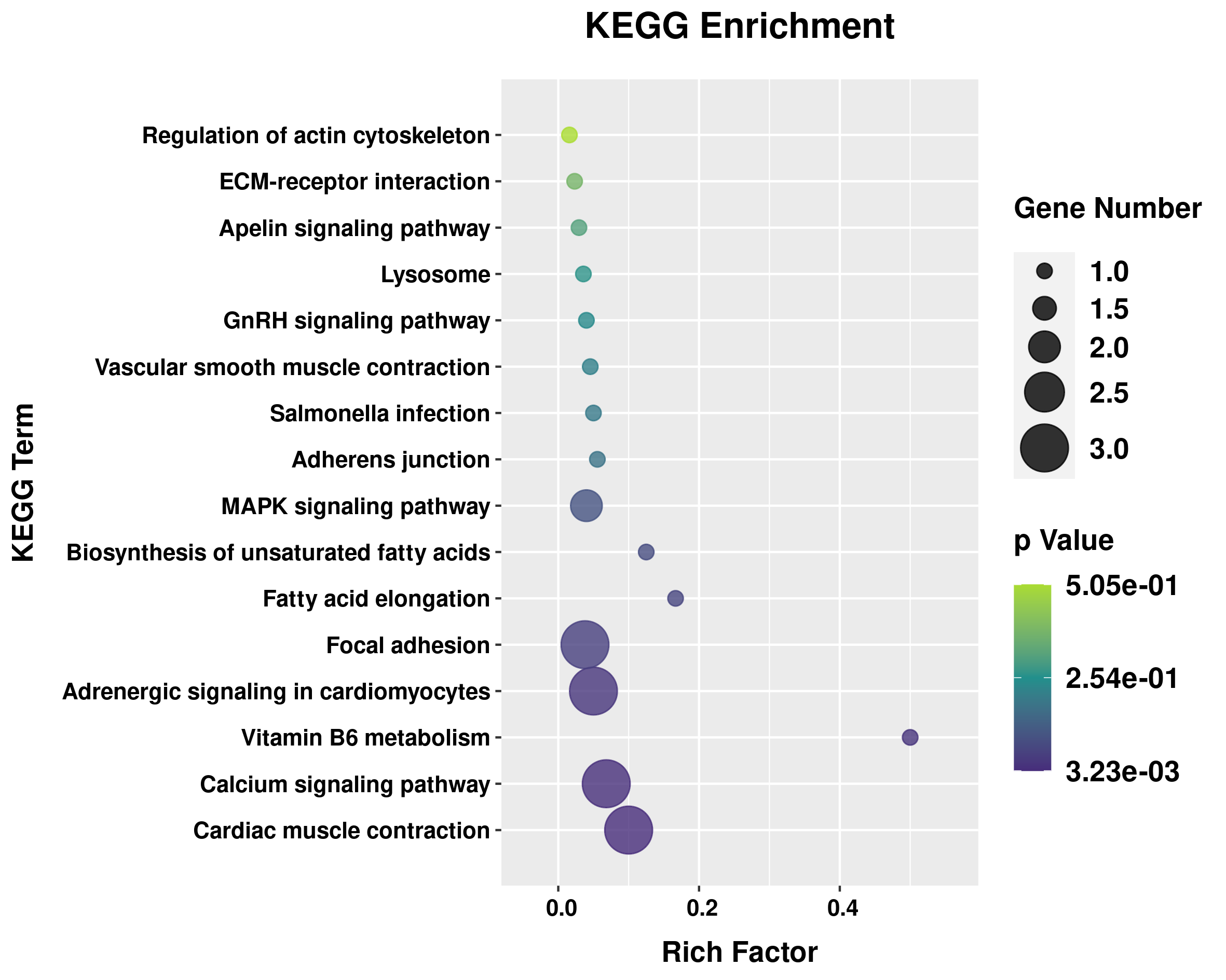

Supplement: Supplementary file 2 — Additional file 2. Fig.s2. Kyoto Encyclopedia of Genes and Genomes (KEGG) analysis of the targets of the total differentially expressed miRNAs (DEMs). Gene number, number of target genes in each pathway. [file 12864_2023_9168_MOESM2_ESM.tiff]

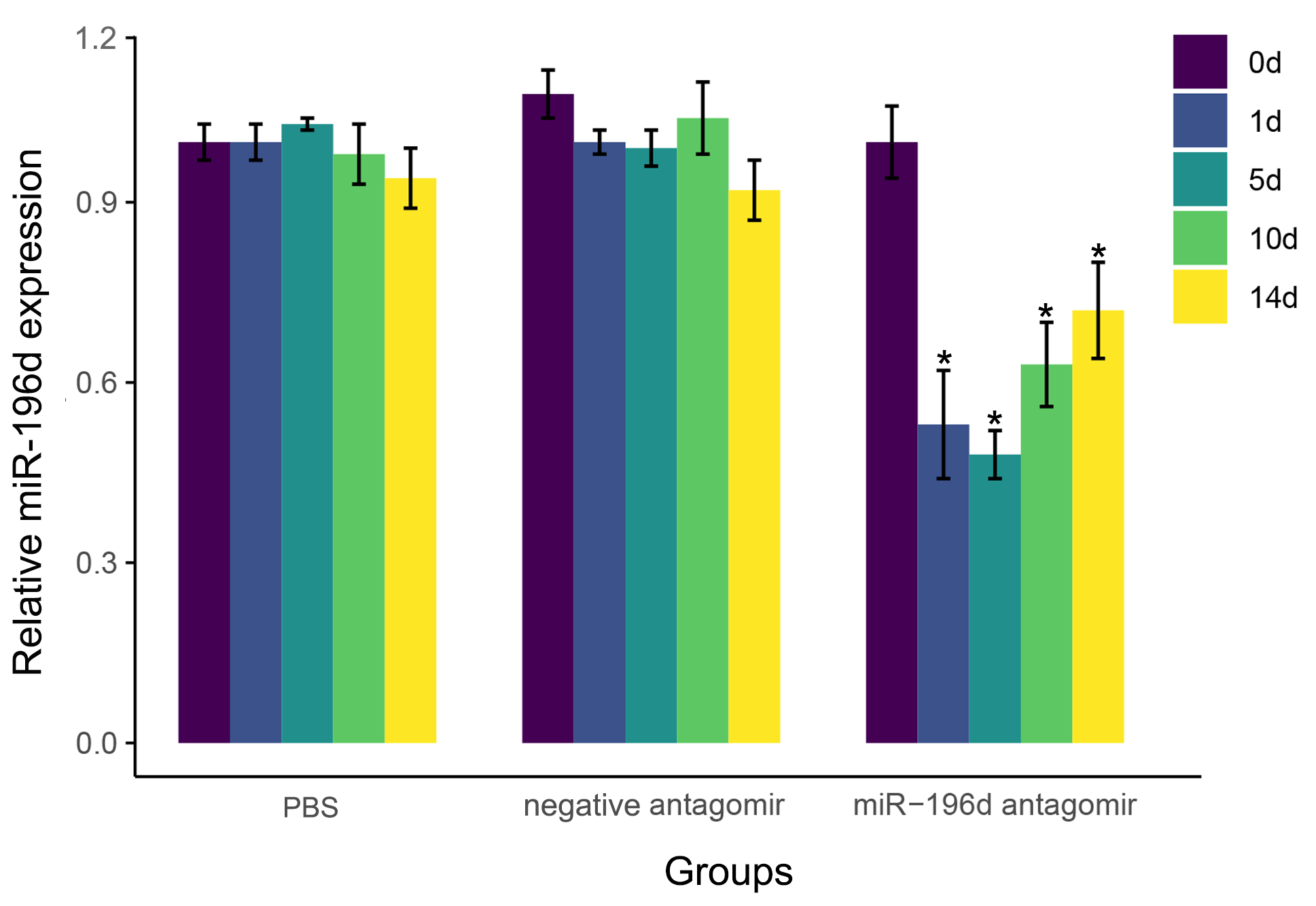

Supplement: Supplementary file 3 — Additional file 3. Fig.s3. Effect of antagomir treatment on miR-196d expression. Fish was injected with miR-206 antagomir, miR-196d antagomir or left untreated, respectively. Asterisk (*) indicates a significant difference compared with the control group. Each sample was analyzed in triplicate. [file 12864_2023_9168_MOESM3_ESM.tif]

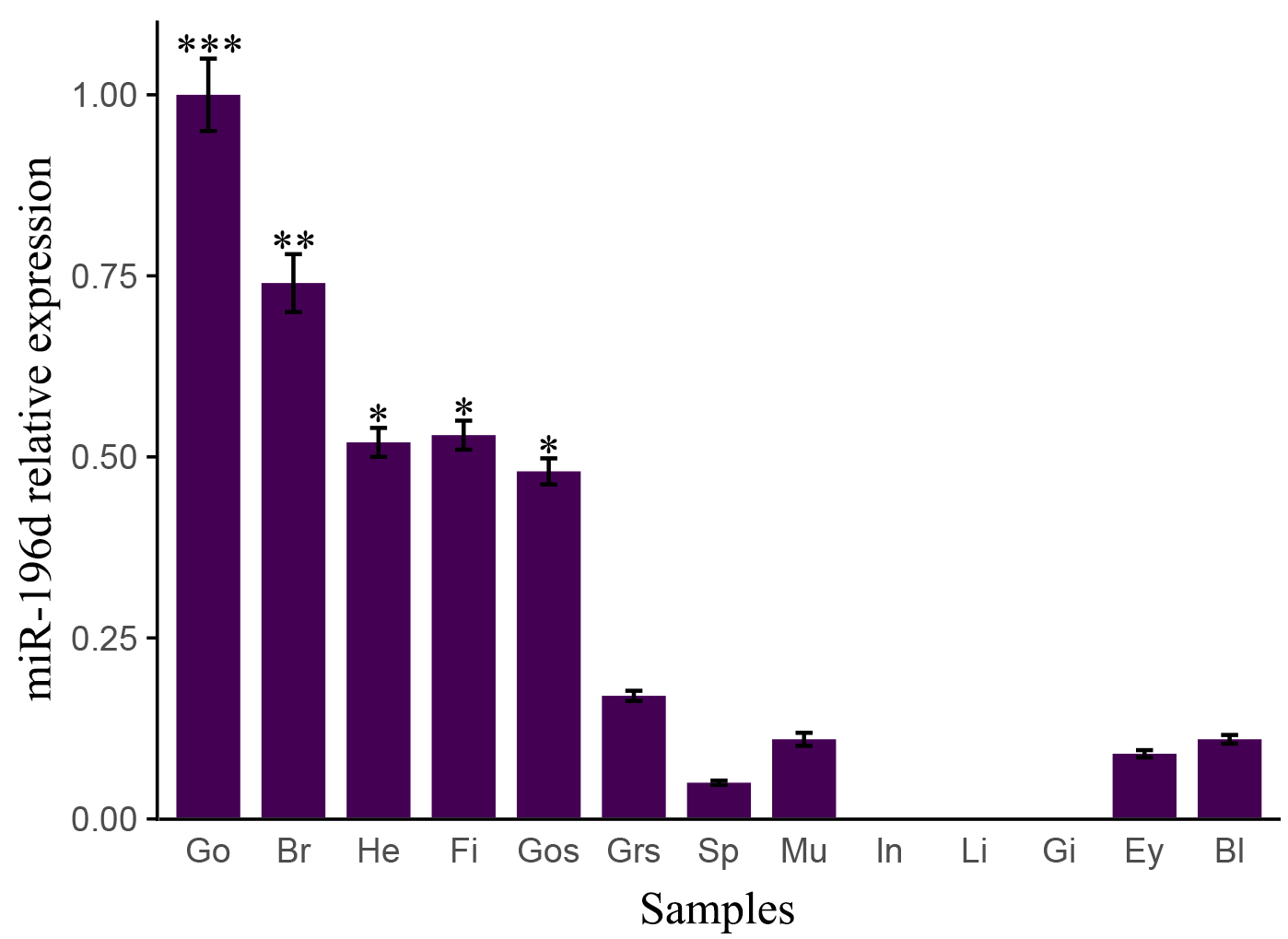

Supplement: Supplementary file 4 — Additional file 4. Fig.s4. Expression pattern of miR-196d in different tissues. Go, gonad; Br, brain; He, heart; Fi, fin; Gos, golden skin; Grs, greenish grey skin; Sp, spleen; Mu, muscle; In, intestine; Li, liver; Gi, gill; Ey, eye; Bl, blood. * p <0.05, ** p <0.01, *** p <0.001. [file 12864_2023_9168_MOESM4_ESM.tif]
